# Selections and Abstracts

**Published:** 1914-05

**Authors:** 


### SELECTIONS AND ABSTRACTS

### THE TYPHUS GERM

By Harry Plotz (A reprint)

Basing my opinion on some theoretical consideration and on previous investigations, I considered it advisable to search for an anerobic organism as the etiologic factor in the acute infectious disease of unknown origin which Brill differentiated from typhus fever. By the use of anaerobic methods in six cases of Brill's disease, I obtained the same organism in five; the case in which the organism was not obtained was investigated only after the crisis.

Inasmuch as studies made during the past few years have shown that Brill's disease is probably a mild form of typhus fever, I decided to apply the same methods to the study of the latter. Through the kindness of Dr. Joseph O'Connell, Health Officer of the Port of New York, to whom I am deeply indebted, I was enabled to study six cases of typhus fever at the height of the disease, and from all of these I recovered an organism that appears to be identical with that isolated from the cases of Brill's disease. A large number of control cases was studied and the organism was absent from each.

The organism is a small Grampositive, pleomorphis bacillus, from 0.9 to 1.93 microns in length, the breadth being from one-fifth to three-fifths of the length. It is not acid-fast, has no capsule, and polar bodies can be demonstrated with appropriate methods. The organism when first isolated grows only anaerobically, but after a time it can be grown aerobically.

Complement-fixation tests were made by Dr. P. K. Olitsky and myself, using the serum of eight cases of typhus fever and antigens made up from organisms obtained both from cases of Brill's disease and typhus fever. Complement-fixation reactions were negative during the course of the disease, but at or after the crises, fixation was found to be present in varying degrees in six out of the eight cases. The antigen made from bacillus obtained from the cases of Brill's disease binds the complement in the same manner as the antigen made from bacillus isolated from the cases of typhus fever. Complement-fixation tests were made in 36 control-cases with absolutely negative results.

Intraperitoneal inoculation of a pure culture of the organism into guinea-pigs produced a rise of temperature in from twenty-four to forty-eight hours, the temperature remaining high four or five days and then dropping by crisis. This corresponds to the reaction seen in guineapigs after inoculation with defibrinated blood from typhus fever patients, except that the incubation period is shorter. Serum from a convalescing typhus fever patient was proved to have bactericidal properties against the organism obtained from Brill's disease and typhus fever.

In a later communication it is proposed to consider the cultural characteristics of the organism, its agglutination reactions, the further results of animal experiments, and cross immunity tests. At the same time the results of studies forming a basis for a possible vaccine prophylaxis and comparative studies of other organisms described by various authors as being found in typhus fever will be reported.

---

### MEETING OF THE AMERICAN MEDICAL EDITORS' ASSOCIATION.

On June 22nd, 9 a. m., the above mentioned Association will meet at the Marlborough-Blenheim Hotel, Atlantic City, N. J., under the Presidency of Dr. E. A. Vander Veer of Albany, N. Y. An unusually attractive program is being prepared. Among the papers are the following:

"Relation of the Medical Press to the Cancer Problem" by Mr. Frederick L. Hoffman, Statistician of the Prudential Ins. Co., Newark, N. J. (by invitation).

"The Things that Count in Medical Practice" by H. Edwin Lewis, M. D., New York.

"Ideal National Medical Journal: What It should be and what It should not be" by W. J. Robinson, M. D., New York.

"Two Problems of the Organization Journal: The Mediocre Paper and the Editoral Department" by Sarah M. Hobson, M. D., Chicago, Ill.

"Medical Journalism as a Local and as a National Proposition" by Thomas S. Blair, M. D., Harrisburg, Pa.

"Medical Books and Journals," by T. D. Crothers, M. D., Hartford, Conn.

"The Medical Periodical and the Scientific Society," by F. H. Garrison, M. D., Washington, D. C.

"Editorial Experiences," by A. L. Benedict, M. D., Buffalo, N. Y.

"The Special Medical Journal," by A. Bassler, M. D., New York.

"The Medical Profession and Its Influence from a Buying Standpoint," by Joseph MacDonald, Jr., M. D., New York.

"The Preparation of the Original Article and the Editors' Latitude," by E. Franklin Smith, M. D., New York.

"He Among You Who is without Sin shall cast the First Stone," by Erwin Reissmann, M. D., Newark.

---

Dr. J. Cheston King, of Atlanta, has been appointed a member of the editorial staff of the Pan-American Medical and Surgical Journal.

---

### COMMON SENSE IN PUBLIC-HEALTH ADMINISTRATION.

By W. C. Rucker, Assistant Surgeon General United States Public Health Service

The amazing fact of epidemiology is its extreme simplicity. Once the causative organism of a disease is known, and the factors concerned in its dissemination have been determined, the measures to be taken to prevent or to eradicate the disease are relatively simple. It may be stated that the greater our ignorance concerning the cause and method of transmission of a disease, the more complicated are our theories as to its epidemiology. Conversely, once the principles which underly the causation and means of spread of a disease become known, we are amazed by the simplicity of the facts. This has produced a twofold action upon the public-health activities of the present day. In the first place, it has made it possible to reduce the incidence of certain communicable diseases with accuracy and precision.

Secondly, it has led large numbers of zealous persons possessed of a few half understood facts to rush into public-health work in its administrative and legislative branches.

In its final analysis, epidemiology is an extremely practical science, and to the mind which has been prepared by proper study and training, it is marvelously easy of application. The technique of prophylactic and eradicative measures is, however, a highly specialized science, which can not be acquired by self-communion and the study of a few text books. The assurance with which the amateur epidemiologist will attack a problem of disease prevention or eradication does not seem in the least amazing to many who would recoil in horror from the thought of an attempted major surgical operation by a sanitarian. With the layman, the case is even worse, and here indeed a little knowledge is an extremely dangerous thing. The physician has a knowledge of the causal elements of disease, and he is also informed as to their means of spread, but he lacks the knowledge of the technique of the application of epidemiological principles. The layman either has none of these, or he has been placed in possession of a few bald facts, without that background of understanding which comes from study and experience.

Take for example the application of the principle that bubonic plague is a rodent disease. The layman may not understand that all that is necessary to control an outbreak or to prevent its recurrence is to thoroughly insulate man against rodents. The layman says: "Let us kill all the rats. We will offer a bounty of 50 cents for female rats and 25 cents for male rats." The epidemiologist says, "No. We will thoroughly rat-proof the habitations of man." Typhoid fever is transmitted by the ingestion of human excrement. The amateur sanitarian says, "We will pass a law forbidding the use of wells." The epidemiologist says, "Very well, but we will also enact laws to the end that sewage may be properly disposed of, the water supply improved, the breeding places of flies destroyed, and the food supplies protected from infection by chronic carriers."

Public-health administration, to be of practical value, must be conducted along logical lines. The prerequisite to a logical course of action is training and the knowledge which comes from it. We must, therefore, expect that until our public-health administrators are thoroughly trained men we can not look for a full measure of common sense in public-health administration. There is at the present time a gradually increasing demand for full-time health officers of proper qualifications, and an attempt is being made by the larger educational institutions to supply this demand by special courses leading to the diploma of public-health. At the present time the salaries offered to such men are not commensurate with the time, labor, and expense which they are obliged to undergo in order to properly fit themselves for the discharge of such duties. The employment of incompetent health officers at a low salary is the worst kind of extravagance. Not only because there will not be a return in efficiency for the moneys so expended, but also because the untrained man will adopt a number of unnecessary and expensive measures which would be avoided by the professional epidemiologist who knows how to perform his duties with accuracy and without lost motion. The untrained health officer establishes a shotgun quarantine, burns tar barrels in the streets, and washes down the houses with bichloride of mercury to stop an outbreak of yellow fever, when the simple measure of Stegomyia destruction will accomplish the desired result.

It should be borne in mind that a wave of public-health enthusiasm is now sweeping over this country, and the American people are being awakened to the realization that we are making a wholly needless sacrifice to preventable disease. Whenever public sentiment is aroused in a particular direction, especially if it be with regard to a subject which requires scientific knowledge and training for proper comprehension, there is great danger that the movement may be characterized by fads and fanaticism. The medical profession of this country is very largely responsible for this sanitary awakening, and " the education of the general public" has become one of the slogans of the campaign. It should not be forgotten that you can not create a soldier by simply giving a man a rifle, and you can not make out of a layman a consistent sanitarian by presenting him with a few predigested facts. Let it be remembered that every action has an equal and opposite reaction, and unless we conduct this educational campaign with common sense and extreme care, the present enthusiasm will be succeeded by an apathetic reversion. Also, do not let us be charmed by the music of our own voices in this educational campaign. Shouting never won a football game, and the speaker sometimes mistakes his own enthusiasm for that of his hearers. The public has been wonderfully patient with us, and while it is extremely desirable that the purposes and aims of sanitation and hygiene should be brought before all citizens, we commit an incalculable error when we endeavor to make them into sanitarians. The best that we can hope will be to popularize the purposes of preventive medicine and to inculcate in the rising generation the principles and practices of personal hygiene. After the age of 25, toothbrush drill very seldom becomes a habit, and we must not expect any marked and widespread change in personal habits as affected by hygiene within the next 15 years.

Almost every time our lawmakers convene they are flooded by a mass of proposed legislation which is to right the sanitary wrongs of the municipality, the State, or the Nation, and unfortunately many of these find their way into the statute books. There is in this country a wealth of sanitary legislation which is impractical of administration and which lacks uniformity and logical basis. The epidemiologist whose business it is to study disease in the light of its prevention has long ago learned that one law in operation is worth ten unenforced laws on the statute books. More than this, an idle law casts discredit upon the legislators who begat it and the officials whose business it is to enforce it. It encourages a disregard for laws which it is desired to enforce, and therefore acts as a general hindrance. Many of the public-health activities in this country would be further advanced today were they not hampered by impractical laws passed by the overzealous. It must be admitted in all justice that the public-health authorities have to a certain extent aided and abetted in the passage of these laws. The enthusiasm of which we have been speaking has led to the formation of a large number of societies having for their object the prosecution of some particular form of public-health activity. The honesty of purpose which has imbued these organizations is unquestioned, but it must be regretfully admitted that their enthusiasm is not always manifested wisely. Too frequently they have led the health officer instead of the health officer leading them, and this has been possible because the health officer has not always been sure in his own mind just what he wanted to do. After the enactment of the law the climax of enthusiasm has been reached, and as it wanes the health officer himself with a weapon in his hands which is so heavy that he can not possibly wield it.

A case in point is the present agitation over track pollution by interstate carriers. From an æsthetic point of view the practice is reprehensible, yet no one has brought forward indisputable evidence that disease is or may be generally spread in this way. Yet the literature on the subject is fairly full, and one author has made the statement that a typhoid carrier traveling from New York to San Francisco by train may infect every mile of roadbed between the two cities. Another writer claims that typhoid fever has a high incidence among traveling salesmen and track employes because they ingest excrement-infected dust. The matter has been carried even further and a bill introduced into one of the State legislatures in order to regulate track pollution. Stripped of its legal verbiage the bill provides that no railway company shall discharge or permit to be discharged from any car carrying passengers any solid or liquid human excreta, either directly or indirectly, within the bounds or on the boundary of any watershed used as a source of public water supply. It further requires that the company shall provide in each car two toilets of such form that they shall thoroughly retain all solid and liquid excreta in a watertight and odorless receptacle. Finally, it is provided that these receptacles shall be emptied, flushed, and thoroughly sterilized or disinfected at least once in every 12 hours of car service, whether in use or not.

Track pollution is undoubtedly a dangerous practice under certain circumstances, and watersheds should always be protected from pollution. The knowledge which we have at hand, however, does not indicate that all track pollution results in human disease. It certainly does not explain the necessity for sterilizing excrement containers which are not in use. What we require first is exact knowledge of the amount of disease spread by track pollution and the mechanism of such spread. Having that, it will be easy enough to legislate sensibly on the question and to put control measures in operation. Premature legislation based on unsupported theories simply reacts to injure the public-health movement.

Two general sets of faults may be found in the sanitary laws of this country. The most common of these is a scatteration of ideas and the loading down of the health officer with more power than he could possibly use. This is just as great a fault as giving him too little power. Frequently a law errs in the opposite direction, and endeavors to be too specific, in which event it becomes the victim of legal quibbles which prove its utter undoing. Most of these laws have been drawn up by amateurs, and even the very wisest professional is sometimes hard put to it to draft a proper law. In this connection, it may be pointed out that the profession of law is becoming highly specialized. We have corporation lawyers for the mining industry, the banking industry, and for the various other classes of corporations. As a matter of fact, there is a specialty for every kind of law from crime to real estate, excepting sanitary law, and there is great need, indeed, for men who can combine with a knowledge of the law a knowledge of the fundamentals of epidemiology.

Too frequently the State or municipality will enact laws giving to the health officer powers to adopt any and all measures for the prevention of the spread of disease, and will appropriate, let us say $1,800 for clerk hire, office rent, and the other purposes of the act. It would be a very good thing if we could have a commission for the determination of sanitary valuation and standardization in order that public-health work might receive the financial support which is essential to efficient administration. Unfortunately, the relation between sanitary authority and sanitary appropriations has never been worked out in a practical way, practical, that is, from the layman's point of view.

### 58,816 MANUFACTURERS TO BE NOTIFIED THAT GUARANTIES AND SERIAL NUMBERS ARE AT AN END MAY 1, 1915.

The Department of Agriculture is sending individual official notices to over 58,000 manufacturers that on May 1, 1915, their guaranties filed under the food and drugs regulations will be stricken from the files and that thereafter the serial numbers assigned to such guaranties must not be used on the label or package of any food or drug. This action is in accordance with the regulations adopted on May 5, 1914, by the Secretaries of the Treasury, Agriculture and Commerce, which abolish the use of the guaranty legend and serial number on food and drugs. The ground for this action was that the legend "Guaranteed by (name of guarantor) under the Food and Drugs Act, June 30, 1906" was understood by many consumers to mean that the Federal Government had passed upon and certified the excellence of the article so labeled, whereas the legend and serial number were merely a guaranty on the part of the manufacturer to his dealer that the manufacturer would assume full legal responsibility for his goods.

In the meantime from the records it appears that 58,816 manufacturers have filed guaranties and obtained serial numbers, the last number issued being 58,816.

The notice advises manufacturers that after May 1, 1915, guaranties should not appear on the label or package but should be incorporated in or attached to the bill of sale, invoice, bill of lading, or other schedule giving the names and quantities of the articles. The guaranty may be printed or stamped on the invoice, and if it is signed in accordance with the new regulations and refers specifically to the goods listed in the invoice or document it covers, it need not contain a detailed description or schedule of the articles.

Manufacturers who are asking permission to file guaranties and obtain serial numbers are being advised that they should attach their guaranty to their invoices and not seek to use the legend or serial number on their labels, as the guaranty and serial number will be withdrawn within a year.

### A SURVEY OF THE PUBLIC HEALTH SITUATION, ATLANTA, GEORGIA.

A REPORT TO THE ATLANTA CHAMBER OF COMMERCE
COMMITTEE ON SOCIAL SURVEY

BY FRANZ SCHNEIDER, JR., SANITARIAN

DEPARTMENT OF SURVEYS AND EXHIBITS, RUSSELL SAGE FOUNDATION.

### I. INTRODUCTORY

In round numbers, Atlanta has 175,000 inhabitants: of these each year three thousand die; three or four thousand more are born alive; while three hundred are born dead. These life and death figures, and the conditions which affect them, mark out the larger aspects of the local public health situation. They have, for our present purposes, four main lines of interest: first, what part of the three thousand deaths represent needless loss of life; second, what machinery exists to prevent such loss; third, what care is taken of the three or four thousand born; and fourth, what new public health machinery, if any, needs to be created?

The presence of unnecessary death implies, of course, the existence in the community of a vastly larger amount of unnecessary sickness; and in attacking the one we are at the same time striking at the other. It is chiefly the prevalence of such sickness and death, and the conditions influencing them, that occupy our attention in a survey of the public health situation. An entirely sufficient argument for the seriousness and importance of such an undertaking is the perfectly obvious and unquestioned relation between public health and public prosperity and happiness.

### II. PREVENTABLE DEATHS IN ATLANTA

Let us consider first the three thousand Atlanta people who die each year; at what age do they perish, and by what causes? Are these deaths unavoidable or preventable? The figures below show the number of deaths which occurred at different age periods in 1910, and cast some light on these questions:

### DEATHS BY AGE GROUPS—ATLANTA, 1910

| Age Groups | Number Deaths | Per cent of |
|---|---|---|
| Under 1 year | 521 | 17.7 |
| Under 5 | 761 | 25.8 |
| 5 to 14 | 105 | 3.6 |
| 15 to 24 | 303 | 10.2 |
| 25 to 50 | 898 | 30.6 |
| 50 and over | 871 | 29.8 |
| Total | 2938 | 100.0 |

Especially notable is the large number of infants dying in their first year—521, or nearly one-fifth of the total mortality; the large number of persons dying between the ages of twenty-five and fifty (the active productive period of life)—898, or nearly one-third of the total; and that 2,067, or over 70 per cent of the deaths, were of persons who had not reached their fiftieth year. These figures show how far short the span of life in Atlanta falls of the ideal of longevity with death by old age; and as ordinarily a large proportion of the population has at fifty only gained the experience which is invaluable for the most efficient work, the figures indicate the seriousness of the economic loss involved.

Turning to the causes of these deaths, and selecting the figures for a number of well known infectious diseases, we have the following array:

### DEATHS FROM CERTAIN PREVENTABLE DISEASES
### ATLANTA—1910

| | | |
|---|---|---|
| Typhoid fever | 78 | |
| Malaria | 4 | |
| Smallpox | 1 | |
| Measles | 14 | |
| Scarlet fever | 8 | |
| Whooping Cough | 16 | |
| Diphtheria | 29 | |
| Tuberculosis: | | |
| Lungs | 282 | |
| Meningitis | 10 | |
| Other | 15 | 457 (15.6% of total mortality.) |
| Diarrhoea (under 2) | 154 | |
| Meningitis | 20 | |
| Pneumonia | 375 | |
| Puerperal fever | 36 | 585 (19.9% of total mortality.) |
| Total | | 1042 (35.5% of total mortality.) |

Notice that the first eight causes, all well-known infectious diseases the preventability of which is generally admitted, give a total of 457 victims, or nearly one-sixth of the year's total mortality; and that adding the figures for the other four causes (infections probably preventable under ideal conditions) we have the astounding total of 1,042 persons—over one-third of the year's entire mortality, dying from preventable diseases. Consider further that these figures represent several times as many persons suffering from non-fatal illness, consider the labor and expense required for their care, and the anguish and suffering caused to their friends and intimates; and it will be admitted that Atlanta offers a large field for public health work. A simple calculation would show that these losses justify large expenditures for preventable measures—expenditures far larger than any that will be advocated in this report.

### 3. EXISTING PREVENTIVE MEASURES

The question now arises as to what machinery is already provided by the city to meet this situation; and whether or not this machinery is equal to the problem at hand.

At present the city entrusts its health work to a Board of Health of ten members; this in turn directs the work of two separate organizations—the Division of Sanitary Inspection, and the Health Department proper. It is with the latter that we are here principally concerned. The Division of Sanitary Inspection is occupied with the collection and disposal of garbage, refuse, and night soil, and with the cleaning of streets and the flushing of sewers: in short, with the engineering aspects of matters relating essentially to public cleanliness and decency. The relation of this division to the Health Department, and its proper position in the municipal organization, will be discussed later; it is sufficient for our present purpose to point out that from the public health standpoint, the most important duty that the Division now performs, is the supervision of privies and the collection of night soil.

### THE HEALTH DEPARTMENT

Turning to the Health Department, which carries on the city's real disease prevention work, we find it in charge of a Health Officer hired and directed by the Board; we find also that it maintains a laboratory, a detention hospital for contagious diseases, and a tuberculosis sanatorium; and that it is engaged in the following branches of work—registration of vital statistics, control of communicable diseases, milk and dairy inspection, meat and market inspection, mosquito reduction, plumbing inspection, and medical relief to the poor. Aside from its hospital staff, the department employs twenty-two persons, and has at its command about $80,000 yearly—a sum which is divided equally between the tuberculosis sanatorium and the department's other work. Omitting from consideration the sum devoted to the sanatorium, the city's health department expenditure is less than twenty three cents per inhabitant per year—a figure which, it must be understood, is considerably below the minimum requirement for a modern, well-rounded, and effective department.

### VITAL STATISTICS

Considering the department's work in detail, let us examine first the registration of vital statistics—the city's system of human bookkeeping. Returns of all marriages, births and deaths must by ordinance be made to the Board of Health; all physicians, accouchers, and midwives must register with it; and burial permits must be obtained from it. Birth must be registered within six days; deaths before removal or burial of the body. These two sets of figures (births and deaths) are those of special hygienic significance; births should be reported promptly if any baby-saving work is to be attempted, and deaths should be reported not only promptly but with accurate and adequate information as to cause and circumstance—as is required in Atlanta by law. The existing six-day limit for birth returns is too lenient, but even this is not enforced. If the excuse be offered that the department makes no use of birth returns, it may be replied that the excuse is simply a confession of neglected opportunity. We have already seen that each year over five hundred Atlanta babies die in their first year of life: in view of the opinions of the best authorities on the preventability of infant deaths, it is entirely probable that a fourth or a third of them could be readily saved. Prompt birth reporting is the essential first step.

The certification of the causes of death is likewise far from satisfactory. An examination of 349 certificates filed during May, 1913, showed the statement of cause to be satisfactory on only 181, while on the remaining 168, or 48 per cent, it was incorrect, incomplete, or indefinite. The statement of occupation of the deceased was stated satisfactorily in 129 instances, was excusably absent in 121 instances; but was unsatisfactorily stated in 99 instances. Statements of the duration of the fatal illness were also commonly lacking. Such certification is not only in violation of the law, but is a testimonial of ignorance and carelessness such as is a serious reproach to the local medical profession. When we find certificates on which the cause of death is merely "Injury during birth," or "Dropsy," or Membranous croup (1 day," or "Choleria Inflammation" or "Acute milk infection (2 months)" with "General Debility (3 months)" as a contributory cause, it is difficult to escape the conclusion that the certifying doctor is still in the dark ages of medicine, and has never even troubled himself to read the directions on the certificate blank. And these defects are by no means limited to Negro doctors. It will be said that the Health Department should refuse to accept such certificates; and it should: but under the present arrangement the certificate is presented by the undertaker, and the health office is usually forced into the unhappy position of holding up a waiting funeral by refusal to accept. The problem should be attacked from both angles; the doctors, as a matter of professional pride and public duty, should seek to improve their certification, while the Health Department should take a firmer attitude in enforcing the provisions of the law. At all events, the problem certainly should be solved, for vital statistics must be the measure of the effectiveness of hygienic endeavor and the guiding index for future developments. Vital statistics should be to the health officer what the balance sheet is to the banker or business man.

Control of Communicable Diseases

The Health Department now requires the reporting of diphtheria, scarlet fever, smallpox, cerebro-spinal meningitis, tuberculosis and typhoid fever; and attempts to prevent their spread. Measles, whooping cough, erysipelas, chicken pox, typhus fever and pellagra were formerly reportable, but were dropped in 1911 at the suggestion of the Fulton County Medical Society. It is difficult to see why all communicable diseases should not be reported to the department. In that direction lies the possibility to applying the known measures to prevent their immediate spread, and the hope of discovering more successful methods of management for the future. The mere signing of a printed post card furnished by the department should certainly be no great hardship to the doctor.

Of the reportable diseases, diphtheria, scarlet fever, smallpox, and cerebro-spinal meningitis are reported to the satisfaction of the department; typhoid fever and tuberculosis are reported in only about half the cases. The laxity with regard to these last two diseases is unfortunate, as they are much more contagious than is usually believed, and as there are a number of very useful things a Health Department can do in the individual cases to prevent the spread of the infection. The department has the authority to prosecute doctors for failures to report these diseases, but hesitates to exercise it.

The department installs quarantine in diphtheria, scarlet fever, and cerebro-spinal meningitis, and sends smallpox to the detention hospital. Only a few of the cases of typhoid are visited, and no steps are taken to follow up tuberculosis; here again the omission is regretable. The regulations for quarantine are in the main satisfactory; and its supervision is as good as can be expected with the small force of two inspectors. Diphtheria is now released ten days after the disappearance of clinical symptoms, although it is generally considered better practice to release only after two successive negative cultures from both throat and nose.

It should be noted that while this branch of the work is in competent hands, it suffers from lack of an adequate staff. With more quarantine inspectors more disease could be investigated and supervised, and more effective isolation could be maintained. Similarly, the isolation hospital is too small, offering accommodations for only thirty persons, and for only two diseases—scarlet fever and diphtheria. No provision is made for cases of measles or erysipelas, or syphilis; nor are there any facilities whatever for negro cases. Laboratory diagnosis in tuberculosis and diphtheria is offered by the department, but again the work is hampered by lack of funds. We are dealing here with preventable diseases at their sources, and many of the essentials are lacking. The city thus neglects a definite opportunity to purchase public health. The question of tuberculosis and the venereal diseases will be discussed at greater length later in the report.

### Milk and Dairy Inspection

The department's program for the improvement of its milk supply is founded on a comprehensive ordinance regulating the condition of dairy farms, depots, etc., and prescribing certain standards for the milk itself, viz: maximum temperature of 55° F., minima of 3.6 per cent and 12 per cent of fats and solids respectively, and a bacterial maximum of 100,000 per cubic centimeter. To carry out the provisions of this law the department has available two dairy inspectors and part of the time of one bacteriologist and an assistant. The effort may be commended as a step in the right direction; but the force is too small to attempt to exercise any real control over the situation. The city now consumes approximately six thousand gallons of milk a day, coming from seven hundred farms, and handled in the city by 235 dealers and forty-one dairies. As in 1912 only 108 samples were analyzed for bacteria, and only 617 dairies were inspected, it will be seen that the city's milk supply is under nothing like continuous supervision. The strength of inspection lies in re-inspection.

As far as the results go, they indicate that the milk supply is in no more than fair condition. The average score of all dairies in 1912 was 50 per cent—a by-no-means too encouraging figure—while the bacteriological counts, though usually in excess of the legal minimum of 100,000, compared favorably with those of the average city. Under these circumstances the Health Officer's statement that he could use two more dairy inspectors to advantage is unquestionably true and moderate; so also is the appeal of the bacteriologist for more assistance. Before leaving the milk situation it should be noted that while the above measures of inspection, if thoroughly carried out, will go a great way toward making the city's milk clean, proper pasteurization is indispensable to safety.

Meat and Market Inspection

The department employs two market inspectors to supervise the condition of markets and perishable foods, and two abatoir inspectors to supervise the slaughtering of cattle. Markets must be licensed and must be screened, the latter requirement prevailing for grocery stores and restaurants, while fruit stores must be screened or have suitable fans. The present market and food inspection does not concern itself with the detection of the more subtle adulterations such as require chemical examination, the department not being equipped to undertake the latter. It is probable also that the present force of two inspectors could be enlarged with advantage.

The work of the two abatoir inspectors is limited to the supervision of killing in three slaughter-houses. The animals are looked over on the hoof and counted; the carcasses are later examined and stamped. It is obvious that when all three houses are killing at the same time, some animals will escape inspection. A very considerable amount of meat is slaughtered in the city —in 1912 close to 70,000 animals, including cattle, calves, hogs, and sheep, and no provision for the inspection of this meat is made beyond the city's partial provision as already described.

### Mosquito Reduction

Between May and September the department carries on a campaign against mosquitoes, employing two inspectors who seek out stagnant water in out-of-the-way places—such as water tanks on buildings, fire buckets and barrels; and four wagon-men who oil the more obvious bodies of water. The Health Officer states that the work has been effective, and believes that providing more men would be money well spent—a conclusion entirely probable when we consider that malaria, from which eight deaths were certified in 1912, is spread only by the mosquito.

### Plumbing Inspection

Nearly a sixth of the department's already scanty appropriation is expended on plumbing inspection, a matter now considered to have very slight hygenic significance. The modern tendency is to place this work with the building department, as the function of the inspector is largely to see that the plumber does an honest job. The inspection protects the citizen's pocket-book rather than his health. Accordingly we may leave this branch of the work with the remark that it is unfortunate that the department must spend for plumbing inspection twice as much as it may spend for the control of communicable diseases.

### Medical Relief

Two city physicians are in the employ of the Health Department, devoting their entire time to the care of the sick poor. If we may judge by their reports there is abundant demand for their services. Here again we may commend the arrangement as a step in the right direction; the utlimate development, however, is a Health Department dispensary, a proposition which will be discussed later in this report.

### IV. DEFECTS IN THE PRESENT ORGANIZATION

### Form of Organization

Let us bring together and consider the various defects in the city's organization for health work which have already been hinted at. The first of these relates to the Board of Health—its size, composition, and duties. The Board is now composed of ten members selected by Council one from each ward, the mayor and chairman of the sanitary committee of Council being members ex-officio. The ward system must be condemned; hygienic procedures must be formulated in accord with science, and not political division or political party. Again, such has been the progress of preventive medicine since the inception of the science of bacteriology and the development of the germ theory of disease, that it is difficult or impossible to obtain as many as ten men in a city of Atlanta's size (or materially larger for that matter) who are competent to formulate and direct the policy of a modern health department.

The board system of health department control is a relic of days in which health work was largely a matter of nuisance abatement and emergency steps in the face of epidemics of small-pox and the like—days when the known procedures were few and simple and when the opinion of a man of general good sense was as valuable in these matters as that of any other. Emphatically those days have now passed; public health work has become intricate and technical; and to leave the responsibility for the progress and administration of a modern health department in the hands of a lay board is about as reasonable as to intrust a similar body with the design and operation of an aeroplane or a submarine. It is now considered wiser to hire a competent specialist in public health work, intrust him with responsibility for the department, and if a board be considered necessary to advise and check him, to have it as small and carefully chosen as possible.

### Other Defects

We have already seen that the local Board of Health directs the work of two separate organizations: the Sanitary Division —occupied with the engineering problems of refuse disposal and street cleaning, and the Health Department proper. This form of organization operates to the distinct disadvantage of the Health Department. The Sanitary Division carries on work which calls for large expenditures of money; and it is but natural that it should hold the attention of the Board to the disadvantage of the comparatively inexpensive Health Department Still the latter directs the city's actual disease prevention work. The operations of the Sanitary Division, having to do with carts and horses and questions of business efficiency—matters of relatively less technical nature and with which its members have had personal experience, is the natural object of interest for the Board, and gives an opportunity for its useful service. The Health Department, on the other hand, with obscure problems of disease and statistics, is beyond the field of experience of the members of the Board. Their control over it must almost inevitably act as a dispiriting handicap.

